# Comparative Evaluation of Ultrasonic and Water Bath Extraction on Bioactive Compounds, Phenolics, Fatty Acids, and Lipid Indices of Raw and Roasted Chia Seeds at Different Temperatures

**DOI:** 10.1002/fsn3.72270

**Published:** 2026-08-21

**Authors:** Isam A. Mohamed Ahmed, Elfadıl E. Babiker, Mehmet Musa Özcan, Belal M. Mohammed, Nurhan Uslu, Mahmoud Younis, Fahad AlJuhaimi, Kashif Ghafoor

**Affiliations:** ^1^ Department of Food Science and Nutrition, College of Food and Agricultural Sciences King Saud University Riyadh Saudi Arabia; ^2^ Faculty of Agriculture, Department of Food Engineering Selcuk University Konya Turkey; ^3^ The School of Agriculture, Food, and Ecosystem Sciences The University of Melbourne Parkville Victoria Australia

**Keywords:** chia seed, lipid indices, phenolics, phytochemicals, ultrasonic and water bath extraction

## Abstract

In this study, the effects of roasting and extraction conditions on the bioactive compounds, antioxidant capacity, fatty acid profile, and lipid quality of chia seed extracts were investigated. The oil content of roasted chia seeds ranged from 25.67% to 29.67% with ultrasonic extraction and from 23.82% to 26.00% with water bath extraction. Total phenolic content varied between 153.61 and 191.67 mg GAE/100 g for ultrasonic extraction and 154.26 and 186.06 mg GAE/100 g for water bath extraction. Total flavonoid content ranged from 48.50 to 64.88 mg/100 g and from 45.45 to 54.07 mg/100 g for ultrasonic and water bath extraction methods, respectively, indicating generally higher extraction efficiency with the ultrasonic method. Roasted chia seeds generally exhibited improved extractable bioactive compounds compared with the control. Both ultrasonic and water bath extraction increased oil yield and the concentrations of total phenolics and total flavonoids, with ultrasonic extraction consistently providing slightly higher values than the water bath. The highest oil yield was obtained by ultrasonic extraction at 40°C, whereas the greatest total phenolic content was achieved at 25°C for 20 min. Total flavonoid content also reached its maximum under ultrasonic extraction at 25°C for 40 min. In raw chia seeds extracted using the ultrasonic bath system, gallic acid and 3,4‐dihydroxybenzoic acid contents ranged from 18.72 to 33.93 mg/100 g and 6.14 to 40.79 mg/100 g, respectively. In roasted chia seeds, catechin and caffeic acid contents ranged from 19.59 to 116.46 mg/100 g and 3.09 to 12.53 mg/100 g, respectively, with the highest values generally obtained at 40°C for 40 min. For individual phenolic compounds, ultrasonic extraction markedly increased gallic acid, 3,4‐dihydroxybenzoic acid, catechin, and caffeic acid contents compared with the control. In general, longer extraction time (40 min) and, for some compounds, higher temperature (40°C) promoted greater phenolic recovery, with the highest catechin and caffeic acid concentrations observed at 40°C for 40 min. Linolenic acid content in oils extracted from roasted chia seeds ranged from 69.32% (control) to 69.71% (40°C/20 min) using ultrasonic extraction, and from 69.46% (25°C/40 min) to 69.81% (40°C/40 min) using water bath extraction. The fatty acid composition was only minimally affected by the extraction method or processing conditions. Linolenic acid remained the predominant fatty acid, with only slight variations among treatments, indicating that neither ultrasonic nor water bath extraction, nor the tested extraction temperature and time, substantially altered the lipid profile. Overall, extraction treatments produced higher values than the control, indicating that both extraction methods enhanced the recovery of these compounds, with ultrasonic extraction showing superior performance.

## Graphical abstract

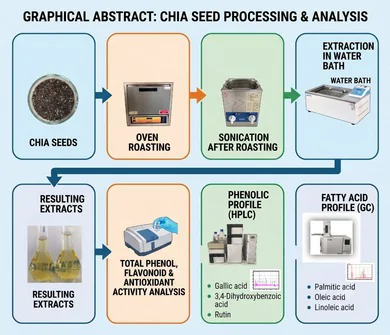

## 1 Introduction

Chia seeds ( *Salvia hispanica* L.; Labiataeae family), an annual herbaceous plant cultivated worldwide (Oliveira‐Alves et al. 2017; Özcan et al. 2019a, 2019b; Ergene and Bingol 2019), contain significant amounts of dietary fiber (34%–40%), protein, and phytochemicals. These components contribute to numerous health benefits, notably antioxidant and cardiovascular effects (Knez Hrnčič et al. 2020; Ghafoor et al. 2020; Islam et al. 2025). Chia seed oil supports human nutrition and health, and its consumption is gaining attention because of its exceptionally high polyunsaturated fatty acid content, exceeding 90% (Ixtaina, Martínez, et al. 2011; Ghafoor et al. 2018). *Salvia hispanica* L. (chia) has attracted increasing attention as a valuable functional food because of its rich composition of bioactive compounds and its well‐documented health‐promoting properties. In particular, chia seeds are an excellent source of dietary fiber, omega‐3 fatty acids, and natural antioxidants, making them an important component of a healthy diet. Research has demonstrated that regular consumption of chia seeds can positively influence lipid metabolism by increasing high‐density lipoprotein cholesterol (HDL‐C) while reducing low‐density lipoprotein cholesterol (LDL‐C), triglycerides, and total cholesterol concentrations. These effects contribute to improved cardiovascular health and highlight the cardioprotective potential of chia seeds (Motyka et al. 2023; Biswas et al. 2023). Flavonoids and tocopherols found in chia seeds have strong antioxidant properties (Hrnčič et al. 2020; Ghafoor et al. 2022). Roasting chia seeds at 120°C for 20 min negatively affected the physicochemical properties of the seeds (Ghafoor et al. 2018). Pulp derived from the chia plant is rich in phenolic constituents exhibiting strong antioxidant and biological activities (Rahman et al. 2017; Dias et al. 2017). Chia seeds are rich in antioxidant compounds, including syringic acid, *p‐*coumaric acid, resveratrol, isorhamnetin, chlorogenic acid, caffeic acid, rutin, myricetin, rosmarinic acid, quercetin, and kaempferol (Ixtaina, Martínez, et al. 2011; Dias et al. 2017; Alcantara et al. 2019; Ghafoor et al. 2020, 2022). Consumption of functional foods has increased significantly because chia seeds provide essential nutrients and phytochemicals that confer health benefits (Al‐Sheraji et al. 2013).

Ultrasound‐assisted extraction is a modern, eco‐friendly, and highly scalable technique that enhances process efficiency and is widely used for obtaining oil and other bioactive compounds from oilseeds (Mwaurah et al. 2020). The mechanism of this extraction method relies on mechanistic events that break down the seed cell walls, increase their permeability, and improve mass transfer rates (Carcel et al. 2012; Mushtaq et al. 2020). To maintain the functional and quality characteristics of low‐oil seeds, different roasting techniques, such as microwave roasting, are commonly employed (Potoˇcnik et al. 2018; Jan et al. 2019; Hatamian et al. 2020). Roasting leads to noticeable changes in the physical appearance, chemical composition, structural attributes, and sensory qualities of oilseeds (Durmaz and Gökmen 2010). Microwave roasting, frequently applied to plant‐based products including edible seeds and nuts, can induce substantial physicochemical alterations in oil and plant products, affecting color, flavor, aroma, fatty acid composition, phytochemicals, and bioactive constituents (Jogihalli et al. 2017; Ghafoor et al. 2022).

Chia seeds are incorporated into flours to enhance the taste, texture, bioactive properties, and functionality of baked goods like bread, biscuits, and chips (Tüter et al. 2025; Islam et al. 2026). They are also used as nutritional supplements in meat, seafood, and other food products (Romankiewicz et al. 2017; Özcan et al. 2019a; Rashid et al. 2021; Islam et al. 2025, 2026). Chia seeds are commercially used as thickeners, emulsifiers, and stabilizers due to their high content of chia gum and mucilage. Additionally, gluten‐free bread made with chia seed flour has been reported to be safe for celiac patients and to contain higher nutritional values, such as vitamins and minerals, than other grains (Özbek and Yeşiilc_ubuk 2018). The present study aimed to evaluate how oil content, total phenolics, total flavonoids, antioxidant capacity, individual phenolic compounds, fatty acid profile, and lipid quality indices differ between raw and roasted chia seeds when extracted using ultrasonic and water bath methods under varying temperatures and extraction durations.

## 2 Material and Methods

### 2.1 Material

In 2025, chia seeds were bought from a seed market in Konya district, Turkey. Afterward, they were stored under cold conditions. Dried seeds were then cleaned using an air screen cleaner to remove dust, immature seeds, and broken seeds. Additionally, any remaining broken kernels were discarded.

### 2.2 Methods

#### 2.2.1 Roasting Process

Chia seeds were evenly distributed in drying trays to a layer about 2 mm thick. They were then heat‐treated in an oven at 175°C for 30 min (Ahmed et al. 2024). Prior to analysis, the samples were ground using a grinder (ARZUM AR1034 Clipper, stainless steel, 150 W motor power, 50 g capacity, Türkiye).

#### 2.2.2 Moisture Content

The KERN & SOHN GmbH infrared moisture analyzer was used for analysis of the moisture contents of chia seed samples. The moisture of raw and roasted chia seeds was 3.67% ± 0.43% and 2.44% ± 0.61%, respectively.

#### 2.2.3 Extraction Procedure

Chia seed samples were extracted according to the method described by Zalewski et al. (2020), with slight modifications. Briefly, 3 g of ground chia seed sample was mixed with 10 mL of a methanol–water solution (70:30, v/v). The mixtures were subjected to ultrasonic‐assisted extraction for 0 min (control), 20 min, or 40 min at either 25°C or 40°C, followed by centrifugation at 6000 rpm for 10 min. The resulting supernatants were evaporated at 40°C, and the dried extracts were reconstituted in 10 mL of methanol. For conventional extraction, the same procedure was followed, except that the samples were extracted using a water bath instead of an ultrasonic bath (Bandelin Sonorex, Germany).

#### 2.2.4 Total Phenolic Amount

The total phenolic content of the extracts was determined using the Folin–Ciocalteu (FC) method as described by Yoo et al. (2004), with slight modifications. Briefly, 1 mL of FC reagent and 10 mL of Na_2_CO_3_ solution were added to the extract, and the mixture was homogenized using a vortex mixer. Deionized water was then added to adjust the final volume to 25 mL. The samples were incubated in the dark for 1 h to allow color development. Subsequently, the absorbance was measured at 750 nm using a spectrophotometer (Shimadzu UVmini 1240, Japan). A calibration curve was established using gallic acid as the standard reference at concentrations ranging from 0 to 200 mg/mL. The total phenolic content was expressed as milligrams of gallic acid equivalents (GAE) per 100 g of sample.

#### 2.2.5 Total Flavonoid Amount

The extract (1 mL) was sequentially mixed with 0.3 mL of NaNO_2_, 0.3 mL of AlCl_3_, and 2 mL of NaOH. The resulting mixture was incubated in the dark for 15 min, after which its absorbance was measured at 510 nm using a spectrophotometer (Shimadzu UVmini 1240, Japan). The results were expressed as mg quercetin equivalents (QE) per 100 g of sample, according to the method described by Hogan et al. (2009).

#### 2.2.6 DPPH Free Radical Scavenging Activity

The free radical scavenging activity of the extracts was evaluated using the DPPH (1,1‐diphenyl‐2‐picrylhydrazyl) assay according to the method described by Lee et al. (1998). Briefly, the extract samples were mixed with 2 mL of a methanolic DPPH solution. Following vigorous shaking, the mixtures were incubated at room temperature for 30 min. The absorbance was then measured at 517 nm using a spectrophotometer (Shimadzu UVmini 1240, Japan). The results were expressed as mmol Trolox equivalents (TE) per kg of sample.

#### 2.2.7 Determination of Phenolic Compounds

The chromatographic separation of phenolic compounds was carried out using a high‐performance liquid chromatography (HPLC) system (Shimadzu, SCL‐10A VP‐Shimadzu, Japan) equipped with a photodiode array (PDA) detector and an Inertsil ODS‐3 column (5 μm particle size; 4.6 × 250 mm). The mobile phase consisted of 0.05% acetic acid in water (solvent A) and acetonitrile (solvent B), which were delivered at a flow rate of 1 mL/min under isocratic/gradient conditions at 30°C. A sample injection volume of 20 μL was used for each analysis. The detection of phenolic compounds was performed at 280 nm using the PDA detector. The gradient elution program was applied as follows: 8% B from 0 to 0.10 min, 10% B from 0.10 to 2 min, 30% B from 2 to 27 min, 56% B from 27 to 37 min, followed by a decrease to 8% B from 37 to 37.10 min and maintained at 8% B from 37.10 to 45 min. The total analysis time for each sample was 60 min.

#### 2.2.8 Oil Extraction

Ground samples (5 g) were placed on filter paper and mixed with 150 mL of petroleum ether in individual flasks. The flasks were then treated in an ultrasonic bath for 20 and 40 min at temperatures of 25°C and 40°C. For conventional extraction, the same procedure was performed using a water bath. After pre‐sonication, the oils were extracted using a Soxhlet apparatus. The solvent was subsequently removed using a rotary vacuum evaporator (Heidolph Laborota 4001, Germany) at 50°C.

#### 2.2.9 Fatty Acid Composition

The oil from chia seeds was esterified according to the method of Multari et al. (2019), and the resulting fatty acid methyl esters were analyzed by gas chromatography (Shimadzu GC‐2010) using a flame‐ionization detector and a capillary column, with both the injector and detector maintained at 260°C.

#### 2.2.10 Calculation

##### 2.2.10.1 Calculated Oxidizability Value (Cox)

The oxidizability ratio values of the chia seed oils depending on lye concentrations were determined according to the method recommended by Fatemi and Hammond (1980).

##### 2.2.10.2 Oleic Desaturation and Linoleic Desaturation Ratio Values

The Oleic Desaturation Ratio (ODR) and Linoleic Desaturation Ratio (LDR) values were determined following the methodology proposed by Pleines and Friedt (1988).

### 2.3 Statistical Analyses

Analysis of variance (ANOVA) was conducted using JMP version 9.0. Data are presented as mean ± standard deviation, calculated with MSTAT C for the independent roasting and sonication conditions.

## 3 Results and Discussion

### 3.1 Physico‐Chemical Properties of Sonicated Raw and Roasted Chia Seeds

Table 1 illustrates the oil content, total phenolic compounds, total flavonoids, and antioxidant capacities of both raw and roasted chia seeds, obtained through ultrasonic bath and water bath extraction methods across different temperatures and extraction durations. The type of extraction, as well as the temperature and duration applied, influenced the oil content and bioactive properties of both raw and roasted chia seeds. The oil yields obtained from raw chia seeds subjected to ultrasonic‐assisted and water bath extraction for 20 min varied from 18.40% at 25°C to 28.00% at 40°C, and from 24.60% at 25°C to 25.00% at 40°C, respectively. The oil contents of raw chia seeds extracted using ultrasonic and water bath methods ranged between 22.40% (control) and 24.80% (40°C), and between 24.80% (control) and 29.80% (40°C), respectively. For roasted chia seeds, the oil yields obtained after 20 min of extraction at different temperatures using ultrasonic‐assisted and water bath extraction techniques were determined to be in the ranges of 25.67% (20 min) to 29.67% (40°C) and 23.82% (control) to 26.00% (40°C), respectively. In raw chia seeds, oil content increased with both temperature and time, with the highest values observed at 40°C for 40 min in both ultrasonic and water bath treatments, except the result observed at 40°C for 20 min in ultrasonic treatment. The highest total phenol quantity was observed in roasted chia seeds extracted using both ultrasonic and water bath methods. The highest total phenolic content observed in roasted chia seeds extracted using both ultrasonic‐assisted extraction and water bath extraction methods can be attributed to the combined effects of roasting and extraction conditions on the release and recovery of phenolic compounds. Roasting may disrupt the cellular structure of chia seeds by breaking down cell walls and weakening the interactions between phenolic compounds and macromolecules such as proteins and polysaccharides, thereby increasing the accessibility of these bioactive compounds during extraction. In addition, thermal treatment can promote the formation of certain phenolic derivatives and enhance the extractability of bound phenolics through structural modifications of the plant matrix (Nicoli et al. 1999; Xu and Chang 2008). Ultrasonic‐assisted extraction further improves phenolic recovery by generating cavitation bubbles that cause mechanical disruption of plant tissues, increasing solvent penetration and facilitating the release of intracellular phenolic compounds. Similarly, water bath extraction provides controlled heating that enhances diffusion and solubility of phenolic compounds in the extraction solvent. Therefore, the higher total phenolic content found in roasted chia seeds obtained by both extraction techniques may result from improved mass transfer, cell wall disruption, and increased liberation of phenolic compounds compared with untreated samples. For raw chia seeds, total phenolic content (TPC) extracted with the ultrasonic bath varied from 103.03 (control) to 159.94 (25°C, 20 min), whereas in the water bath system, TPC ranged from 102.76 (control) to 162.69 mg GAE/100 g (25°C, 40 min) (Table 2). Additionally, roasted chia seeds extracted under different temperatures and times in both systems showed TPC values between 153.61 (control) and 191.67 mg GAE/100 g (25°C, 20 min) for the ultrasonic system, and 154.26 (control) and 186.06 mg GAE/100 g (25°C, 40 min) for the water bath system (Table 3). While total flavonoid contents of the raw chia seeds obtained by ultrasonic bath extraction method are assessed between 43.88 (control) and 72.74 mg/100 g (25°C/20 min), total flavonoid amounts of the raw chia seeds obtained by water bath system were assessed to be between 44.36 (control) and 82.36 mg/100 g (25°C/40 min) (Table 2). Also, total flavonoid amounts (TFC) of the roasted chia seeds obtained by ultrasonic and water bath extraction methods were demonstrated to be between 48.50 (40°C/20 min) and 64.88 mg/100 g (25°C/40 min) to 45.45 (40°C/20 min) and 54.07 mg/100 g (25°C/40 min), respectively. The antioxidant activities of raw chia seeds extracted by ultrasonic and water bath extraction were determined to be 6.87 (25°C/40 min) and 6.92 mmol TE/kg (40°C/40 min) and 6.88 (25°C/40 min) and 6.91 mmol TE/kg (40°C/40 min), respectively. While antioxidant capacities of the roasted chia seeds extracted by ultrasonic bath extraction method are established between 6.84 (40°C/40 min) and 7.04 mmolTE/kg (control), antioxidant activities of the roasted chia seeds were described to be between 6.84 (25°C/20 min) and 6.88 mmolTE/kg (control). The antioxidant activity values of raw and roasted chia seeds extracted by both systems were comparable, with no significant differences observed. Roasting process did not result in a consistent increase in the antioxidant activity of chia seeds; in fact, antioxidant activity values remained within a narrow range across both raw and roasted samples regardless of the sonication parameters. In this case, it is thought that the applied processes did not have a significant effect on antioxidant activity values. The control sample exhibited a total phenolic content of 2.55 mg GAE/g, while chia seeds roasted at 90°C and 120°C showed decreased values of 2.34 mg GAE/g and 2.14 mg GAE/g, respectively (Al‐Juhaimi et al. 2024). Correspondingly, the total flavonoid contents were 13.71 mg CE/g for seeds roasted at 90°C and 12.91 mg CE/g for those roasted at 120°C (Al‐Juhaimi et al. 2024). Similarly, Ghafoor et al. (2018) reported total phenolic contents of 3.07 mg GAE/g in raw chia seeds, which increased to 3.43 mg GAE/g upon roasting. Marineli et al. (2014) determined that chia seeds contained 0.94 mg GAE/g of total phenolics. Additionally, Ghafoor et al. (2020) observed total phenol levels of 0.98 mg GAE/100 g in control samples, which declined to 0.91 and 0.88 mg GAE/100 g following roasting at 90°C and 120°C, respectively. Ahmed et al. (2024) reported that the total phenolic content of roasted chia seeds ranged from 186.59 mg GAE/100 g in the control to 429.29 mg GAE/100 g after 20 min of roasting, while total flavonoid content varied from 810.00 mg/100 g at 10 min to 903.33 mg/100 g at 5 min of roasting. Furthermore, Martínez‐Cruz and Paredes‐López (2014) determined a DPPH antioxidant capacity of 68.83%. The pronounced antioxidant activity of chia seeds is likely attributable to their high content of phenolic compounds, including phenolic acids, isoflavones, and anthocyanins (Martínez‐Cruz and Paredes‐López 2014). Total phenolic content varies with cultivar and study. For example, values of 0.88–0.92 mg GAE/g have been reported for the Sinaloa and Jalisco cultivars (Reyes‐Caudillo et al. 2008), and 1.64 mg GAE/g (Martínez‐Cruz and Paredes‐López 2014). Roasting has been shown to significantly reduce antioxidant activity, as demonstrated by Ghafoor et al. (2020), who observed a progressive decline from 88.27% in unroasted seeds to 85.76%, 79.57%, 48.44%, and 31.23% at 90°C, 120°C, 150°C, and 180°C, respectively. In agreement, Ahmed et al. (2024) reported that roasting chia seeds at 120°C for 10–20 min in a Teflon pan resulted in a marked decrease in their antioxidant capacity. The oil content of chia seeds was significantly influenced by factors such as the seed's state (raw or roasted), the processing method, temperature, and the duration of treatment (*p* < 0.05). In raw chia seeds, oil content increased with both temperature and time, with the highest values observed at 40°C for 40 min in both ultrasonic and water bath treatments, except the result observed at 40°C for 20 min in ultrasonic treatment. Ultrasonic processing resulted in greater oil extractability than water bath treatment under comparable conditions, which can be attributed to acoustic cavitation effects that promote cell wall disruption and enhance solvent penetration. Roasted chia seeds exhibited significantly higher oil contents than raw seeds across all treatments. This increase is likely due to structural weakening of the seed matrix during roasting, facilitating oil release during subsequent processing. However, the enhancement of oil yield at higher temperatures was often accompanied by a reduction in bioactive compounds, indicating a temperature‐dependent trade‐off between oil extraction efficiency and phytochemical retention. Both ultrasonic and water bath treatments significantly increased total phenolic content compared with control samples (*p* < 0.05). In raw chia seeds, the highest TPC values were observed at 25°C, particularly after 40 min of water bath treatment, suggesting that mild processing conditions promote phenolic release without inducing thermal degradation. Ultrasonic treatment at 25°C also resulted in substantial increases in TPC roasted chia, reflecting the effectiveness of cavitation in liberating bound phenolic compounds. In contrast, increasing the processing temperature to 40°C generally resulted in reduced TPC in both raw and roasted seeds. This decrease may be attributed to oxidative degradation or polymerization of thermolabile phenolic compounds under prolonged thermal exposure. Roasted chia seeds displayed higher baseline TPC than raw seeds, indicating that roasting may enhance phenolic availability through matrix modification or the formation of Maillard reaction‐derived phenolics. Also, the significant increase in total phenolic content (TPC) of roasted chia seeds after ultrasonic treatment applied at 25°C can be attributed to the enhanced mass transfer and disruption of the seed matrix caused by ultrasonic cavitation. During ultrasonication, the formation and collapse of microscopic bubbles generate localized shear forces, microjets, and turbulence, which break down cell wall structures and improve the release of bound phenolic compounds from the plant tissue. Although roasting can promote the formation of some antioxidant compounds through thermal reactions, excessive heat may also cause degradation of phenolics; therefore, the application of ultrasound at a mild temperature (25°C) provides an additional extraction mechanism without inducing significant thermal degradation. In chia seeds, many phenolic compounds are associated with cell wall components, and ultrasonic treatment facilitates their migration into the extract, resulting in higher measurable TPC values. Similar improvements in phenolic recovery after ultrasound‐assisted extraction have been reported for various plant materials, where cavitation‐induced structural disruption increased the accessibility of bioactive compounds (Chemat et al. 2017; Tiwari 2015). Therefore, the combination of roasting‐induced structural modification and ultrasound‐assisted release mechanisms likely explains the observed increase in TPC in roasted chia seeds. Total flavonoid content (TFC) followed trends similar to those observed for TPC but exhibited greater sensitivity to temperature. The highest TFC values were recorded at 25°C for extended treatment durations, particularly under ultrasonic processing for raw seeds and water bath treatment for roasted seeds. These findings suggest that flavonoids are effectively released under mild conditions but are susceptible to degradation at elevated temperatures. At 40°C, TFC decreased significantly regardless of processing method, confirming the thermal instability of flavonoid compounds. Although roasting increased initial flavonoid levels, prolonged exposure to higher temperatures during post‐roasting processing resulted in partial losses. Antioxidant activity showed relatively limited variation across treatments compared with TPC and TFC. The antioxidant activity values of raw and roasted chia seeds extracted by both systems were comparable, with no significant differences observed. Roasting process did not result in a consistent increase in the antioxidant activity of chia seeds; in fact, antioxidant activity values remained within a narrow range across both raw and roasted samples regardless of the sonication parameters. In this case, it is thought that the applied processes did not have a significant effect on antioxidant activity values. Roasting process did not result in a consistent increase in the antioxidant activity of chia seeds; in fact, antioxidant activity values remained within a narrow range across both raw and roasted samples regardless of the sonication parameters. Slight decreases in antioxidant activity were observed at higher temperatures and longer processing times, particularly when accompanied by reductions in TPC and TFC. This indicates that phenolic compounds play a primary role in antioxidant capacity, although other non‐phenolic antioxidants may contribute to maintaining relatively stable activity levels.

**TABLE 1:** Bioactive properties of raw and roasted chia seeds extracted by ultrasonic and water bath methods.

Sample	Process	Temperature	Time (Min)	Oil content (%)	Total phenolic content (mgGAE/100 g)	Total flavonoid content (mgQE/100 g)	Antioxidant activity (mmolTE/kg)
Raw chia seed	Control	—	—	22.40 ± 0.79d	103.03 ± 2.69e	43.88 ± 3.68e	6.90 ± 0.03
	Ultrasonic bath	25°C	20	18.40 ± 0.65e	159.94 ± 1.88a	72.74 ± 6.62a	6.90 ± 0.03
		40°C		28.00 ± 0.99a	156.27 ± 0.14b	48.60 ± 1.67d	6.91 ± 0.04
		25°C	40	22.60 ± 0.80c	153.44 ± 2.54c	66.31 ± 5.64b	6.87 ± 0.07
		40°C		24.60 ± 0.87b	147.93 ± 5.44d	55.40 ± 1.62c	6.92 ± 0.01
	Control	—	—	24.80 ± 0.88d	102.76 ± 1.31e	44.36 ± 0.76e	6.90 ± 0.01
	Water‐bath	25°C	20	24.60 ± 0.87e	142.69 ± 4.67b	74.45 ± 4.51b	6.90 ± 0.02
		40°C		25.00 ± 0.88c	120.76 ± 5.05d	51.55 ± 3.79d	6.92 ± 0.00
		25°C	40	29.60 ± 1.05ab	162.69 ± 2.13a	82.36 ± 3.58a	6.88 ± 0.03
		40°C		29.80 ± 1.05a	128.61 ± 2.04c	53.07 ± 2.81c	6.91 ± 0.01
Roasted chia seed	Control	—	—	25.94 ± 0.59c	153.61 ± 5.05e	54.50 ± 2.00d	7.04 ± 0.06
	Ultrasonic bath	25°C	20	25.67 ± 0.54d	191.67 ± 2.81a	57.21 ± 2.92b	6.98 ± 0.05
		40°C		29.67 ± 0.63b	164.19 ± 4.74d	48.50 ± 0.57e	6.93 ± 0.08
		25°C	40	25.00 ± 0.53e	184.46 ± 2.77b	64.88 ± 4.13a	6.85 ± 0.05
		40°C		31.67 ± 0.67a	182.97 ± 5.18c	55.69 ± 2.38c	6.84 ± 0.03
	Control	—	—	23.82 ± 0.57e	154.26 ± 5.52d	50.36 ± 6.08c	6.88 ± 0.01
	Water‐bath	25°C	20	25.33 ± 0.54d	173.82 ± 3.61b	53.26 ± 0.93b	6.85 ± 0.01
		40°C		26.00 ± 0.55c	152.49 ± 5.15e	45.45 ± 1.25e	6.84 ± 0.02
		25°C	40	28.00 ± 0.59b	186.06 ± 0.14a	54.07 ± 1.12a	6.86 ± 0.01
		40°C		30.67 ± 0.86a	157.80 ± 3.46c	48.60 ± 1.59d	6.86 ± 0.01

*Note:* Values within each column followed by different letters are significantly different (*p* < 0.05) within each processing method independently.

**TABLE 2:** Phenolic compounds of raw and roasted chia seeds extracted by ultrasonic and water bath methods.

Phenolic compounds (mg/100 g)	Ultrasonic bath				
	Control	25°C/20 min	40°C/20 min	25°C/40 min	40°C/40 min
Gallic acid	18.72 ± 1.21d	31.42 ± 2.23b	24.53 ± 2.08c	33.93 ± 2.63a	24.09 ± 1.05c
3,4‐Dihydroxybenzoic acid	6.14 ± 0.90e	10.23 ± 1.44d	32.18 ± 1.54b	40.79 ± 1.51a	31.33 ± 3.38c
Catechin	34.77 ± 0.63d	76.24 ± 4.10a	26.86 ± 0.87e	50.19 ± 2.35b	41.75 ± 1.10c
Caffeic acid	0.69 ± 0.30e	22.97 ± 1.28a	4.08 ± 0.07d	9.69 ± 0.98b	7.33 ± 2.65c
Syringic acid	0.67 ± 0.10e	9.18 ± 0.42b	3.26 ± 0.29c	12.28 ± 1.42a	2.83 ± 0.91d
Rutin	2.23 ± 0.44 cd	40.26 ± 3.05a	2.53 ± 0.84c	11.51 ± 0.50b	0.55 ± 0.06e
*p*‐Coumaric acid	0.51 ± 0.09c	7.46 ± 0.80a	0.54 ± 0.17c	0.87 ± 0.08b	0.57 ± 0.09c
Ferulic acid	1.31 ± 0.33b	9.06 ± 1.07a	0.35 ± 0.08d	0.50 ± 0.06c	0.53 ± 0.10c
Resveratrol	0.23 ± 0.05e	2.29 ± 0.03a	0.38 ± 0.11d	0.67 ± 0.03b	0.48 ± 0.06c
Quercetin	0.81 ± 0.05d	8.30 ± 0.44a	1.78 ± 0.37b	1.70 ± 0.31b	1.11 ± 0.27c
Cinnamic acid	0.10 ± 0.02c	0.45 ± 0.13a	0.10 ± 0.03c	0.11 ± 0.02c	0.32 ± 0.08b
Kaempferol	5.05 ± 0.21e	97.19 ± 2.81a	67.00 ± 0.58d	90.30 ± 0.76b	85.91 ± 4.63c

Phenolic compounds (mg/100 g)	Water‐bath				
	Control	25°C/20 min	40°C/20 min	25°C/40 min	40°C/40 min
Gallic acid	1.92 ± 0.15e	23.07 ± 2.46a	22.62 ± 1.98b	12.05 ± 2.29d	21.49 ± 1.04c
3,4‐Dihydroxybenzoic acid	5.03 ± 1.62e	16.18 ± 1.74c	15.33 ± 0.92d	21.85 ± 0.95b	44.74 ± 0.38a
Catechin	5.89 ± 0.38e	47.60 ± 0.76b	19.67 ± 1.93c	15.76 ± 1.88d	50.61 ± 1.52a
Caffeic acid	0.12 ± 0.02e	11.08 ± 0.94a	2.02 ± 0.20d	4.76 ± 2.23c	9.49 ± 0.53b
Syringic acid	0.44 ± 0.15e	9.05 ± 1.56a	0.79 ± 0.11d	3.01 ± 1.51c	7.19 ± 1.94b
Rutin	0.83 ± 0.13e	5.33 ± 1.03b	3.57 ± 0.81d	4.65 ± 1.18c	45.20 ± 2.98a
*p*‐Coumaric acid	0.17 ± 0.04d	2.21 ± 0.96a	1.34 ± 0.14c	1.66 ± 0.56b	1.30 ± 0.24c
Ferulic acid	0.41 ± 0.14de	4.36 ± 0.78a	0.58 ± 0.29d	1.65 ± 0.85c	3.26 ± 0.67b
Resveratrol	0.43 ± 0.03c	0.52 ± 0.15b	0.73 ± 0.11a	0.20 ± 0.03e	0.35 ± 0.02d
Quercetin	0.57 ± 0.11d	1.19 ± 0.15c	1.47 ± 0.23b	0.41 ± 0.10e	1.54 ± 0.18a
Cinnamic acid	7.97 ± 2.27c	0.25 ± 0.07d	7.92 ± 2.26c	12.67 ± 3.63b	15.83 ± 0.43a
Kaempferol	66.99 ± 3.22c	83.40 ± 0.16b	66.13 ± 1.16c	104.64 ± 2.16a	15.91 ± 1.28d

*Note:* Values within each row followed by different letters are significantly different at *p* < 0.05.

**TABLE 3:** Phenolic compounds of raw and roasted chia seeds extracted by ultrasonic and water bath methods.

Phenolic compounds (mg/100 g)	Ultrasonic bath				
	Control	25°C/20 min	40°C/20 min	25°C/40 min	40°C/40 min
Gallic acid	23.90 ± 2.22*b	14.69 ± 0.83d	14.23 ± 0.69de	20.07 ± 0.62c	26.96 ± 1.83a
3,4‐Dihydroxybenzoic acid	19.08 ± 1.78b**	6.93 ± 0.80d	4.32 ± 0.77e	29.12 ± 1.96a	18.42 ± 0.85c
Catechin	52.50 ± 0.57c	57.03 ± 0.53b	19.59 ± 0.58e	32.86 ± 1.25d	116.46 ± 8.34a
Caffeic acid	3.69 ± 0.79d	3.09 ± 1.01de	8.85 ± 0.91c	10.31 ± 0.13b	12.53 ± 2.30a
Syringic acid	6.82 ± 0.02c	2.22 ± 0.60de	2.34 ± 0.21d	17.38 ± 1.72a	11.94 ± 2.18b
Rutin	35.67 ± 3.42a	7.19 ± 0.21d	4.52 ± 0.24e	30.11 ± 0.04b	22.60 ± 1.99c
*p*‐Coumaric acid	1.96 ± 0.66c	1.45 ± 0.36d	1.20 ± 0.32e	5.61 ± 0.86a	4.19 ± 1.08b
Ferulic acid	0.47 ± 0.03e	0.85 ± 0.30d	1.67 ± 0.61c	6.55 ± 0.14ab	6.68 ± 2.38a
Resveratrol	1.46 ± 0.48c	0.24 ± 0.09e	0.40 ± 0.04d	3.55 ± 0.06b	5.10 ± 0.47a
Quercetin	6.84 ± 0.30c	1.66 ± 0.33e	2.18 ± 0.44d	7.04 ± 0.04b	15.12 ± 1.02a
Cinnamic acid	1.08 ± 0.08d	1.16 ± 0.12c	0.80 ± 0.10e	1.24 ± 0.21b	2.40 ± 0.40a
Kaempferol	1.01 ± 0.38e	37.81 ± 0.46c	25.84 ± 1.89d	42.68 ± 2.60b	53.02 ± 3.37a

Phenolic compounds (mg/100 g)	Water‐bath				
	Control	25°C‐20 min	40°C‐20 min	25°C‐40 min	40°C‐40 min
Gallic acid	21.41 ± 1.22a	21.14 ± 1.61a	19.01 ± 1.54b	18.25 ± 1.08c	17.91 ± 2.49d
3,4‐Dihydroxybenzoic acid	33.04 ± 0.88b	28.08 ± 1.45c	23.37 ± 4.48d	35.12 ± 0.92a	21.01 ± 0.76e
Catechin	84.34 ± 0.91e	292.57 ± 0.58a	261.08 ± 7.78b	235.89 ± 8.55c	226.47 ± 0.03d
Caffeic acid	16.31 ± 1.84a	8.03 ± 1.16d	13.44 ± 1.94b	6.96 ± 0.23e	8.39 ± 0.46c
Syringic acid	9.44 ± 1.12 cd	22.96 ± 2.10a	19.05 ± 1.29b	6.50 ± 1.05e	9.74 ± 3.25c
Rutin	82.69 ± 3.24a	45.13 ± 2.90b	45.27 ± 3.15b	31.32 ± 4.22c	16.69 ± 3.47d
*p*‐Coumaric acid	9.85 ± 0.49a	5.18 ± 1.49d	6.11 ± 0.83c	9.27 ± 1.40b	3.45 ± 1.51e
Ferulic acid	2.57 ± 1.01e	3.96 ± 0.69c	9.62 ± 0.59a	8.14 ± 0.99b	3.68 ± 1.81 cd
Resveratrol	4.64 ± 0.29a	3.88 ± 0.96b	1.89 ± 0.47c	1.84 ± 0.76c	1.03 ± 0.41d
Quercetin	7.65 ± 1.44d	8.90 ± 1.31c	10.51 ± 3.09b	12.56 ± 0.31a	5.57 ± 2.17e
Cinnamic acid	2.82 ± 0.83b	1.62 ± 0.35e	1.85 ± 0.34c	1.70 ± 0.40d	8.08 ± 1.28a
Kaempferol	66.13 ± 0.84b	68.98 ± 1.65a	41.51 ± 2.11c	41.33 ± 2.79c	8.19 ± 0.41d

*Note:* *, Standard deviation; **, Values within each row followed by different letters are significantly different at *p* < 0.05.

### 3.2 Phenolic Compounds of Raw Chia Seeds Extracted With Ultrasonic and Water Bath Systems

The phenolic composition of raw and roasted chia seeds, obtained using both ultrasonic and water bath extraction methods under different thermal and temporal conditions, is summarized in Tables 2 and 3. Roasted chia seeds generally have higher phenolic compound content compared to unroasted seeds. The major phenolic compounds identified in raw chia seed extracts, obtained using both extraction methods, were gallic acid, 3,4‐dihydroxybenzoic acid, catechin, rutin, cinnamic acid (with the exception of extracts obtained via ultrasonic bath), and kaempferol. Quantitative analysis indicated that the concentrations of gallic acid and 3,4‐dihydroxybenzoic acid in extracts produced by ultrasonic bath extraction ranged from 18.72 mg/100 g (control) to 33.93 mg/100 g (25°C, 40 min) and from 6.14 mg/100 g (control) to 40.79 mg/100 g (25°C, 40 min), respectively, demonstrating a notable variation in phenolic content depending on extraction conditions (Table 2). While catechin contents of the raw chia seeds are established between 26.86 (40°C/25 min) and 76.24 mg/100 g (25°C/20 min), caffeic acid amounts of the raw chia seeds were characterized to be between 0.69 (control) and 22.97 mg/100 g (25°C/20 min). Rutin and kaempferol contents of the raw chia seeds were described to be between 0.55 (40°C/40 min) and 40.26 mg/100 g (25°C/20 min) to 5.04 (control) and 97.19 mg/100 g (25°C/20 min), respectively (Table 2). Gallic and 3,4‐dihydroxybenzoic acid amounts of the raw chia seeds extracted by water bath extraction method were assayed to be between 1.92 (control) and 23.07 mg/100 g (25°C/20 min) to 5.03 (control) and 44.74 mg/100 g (40°C/40 min), respectively. Catechin and caffeic acid contents of the raw chia seeds obtained by water bath system were characterized to be between 5.89 (control) and 50.61 mg/100 g (40°C/40 min) to 0.12 (control) and 11.08 mg/100 g (25°C/20 min), respectively (Table 2). While syringic acid amounts of the raw chia seeds obtained by water bath system are assayed between 0.44 (control) and 9.05 (25°C/20 min), rutin amounts of the raw chia seeds were described to be between 0.83 (control) and 45.20 mg/100 g (40°C/40 min). The concentrations of cinnamic acid and kaempferol in raw chia seeds, extracted via a water bath at varying temperatures and durations, were found to range from 0.25 mg/100 g (25°C, 20 min) to 15.83 mg/100 g (40°C, 40 min) and from 15.91 mg/100 g (40°C, 40 min) to 104.64 mg/100 g (25°C, 40 min), respectively. Gallic acid and 3,4‐dihydroxybenzoic acid amounts of the roasted chia seeds obtained by ultrasonic bath extraction method were assessed to be between 14.23 (40°C/20 min) and 26.96 mg/100 g (40°C/40 min) to 4.32 (40°C/20 min) and 29.12 mg/100 g (25°C/40 min) (Table 3). While catechin quantities of the roasted chia seeds obtained by the ultrasonic bath method are described to be between 19.59 (40°C/20 min) and 116.46 mg/100 g (40°C/40 min), caffeic acid amounts of the roasted chia seeds were assayed to be between 3.09 (25°C/20 min) and 12.53 mg/100 g (40°C/40 min). Syringic acid and routine amounts of the roasted chia seeds were determined to be between 2.22 (25°C/20 min) and 17.38 mg/100 g (25°C/40 min) to 4.52 (40°C/20 min) and 35.67 mg/100 g (control), respectively (Table 3). The amounts of quercetin and kaempferol in roasted chia seeds extracted using the ultrasonic system were found to range from 1.66 mg/100 g (25°C/20 min) to 15.12 mg/100 g (40°C/40 min) and from 1.01 mg/100 g (control) to 53.02 mg/100 g (40°C/40 min), respectively. For roasted chia seeds extracted by the water bath system, gallic acid and 3,4‐dihydroxybenzoic acid contents varied between 17.91 mg/100 g (40°C/40 min) and 21.41 mg/100 g (control) and between 21.01 mg/100 g (40°C/40 min) and 35.12 mg/100 g (25°C/40 min), respectively. Catechin levels in water bath–extracted roasted chia seeds were determined to be 84.34 mg/100 g (control) and 292.57 mg/100 g (25°C/20 min), while caffeic acid amounts range from 6.96 mg/100 g (25°C/40 min) to 16.31 mg/100 g (control). Syringic acid and rutin concentrations were found to range from 6.50 mg/100 g (25°C/40 min) to 22.96 mg/100 g (25°C/20 min) and from 16.69 mg/100 g (40°C/40 min) to 82.69 mg/100 g (control), respectively. Quercetin amounts in water bath–extracted seeds ranged from 5.57 mg/100 g (40°C/40 min) to 12.56 mg/100 g (25°C/40 min), while cinnamic acid levels are assayed between 1.62 mg/100 g (25°C/20 min) and 8.08 mg/100 g (40°C/40 min). Kaempferol amounts were identified to range from 8.19 mg/100 g (40°C/40 min) to 68.98 mg/100 g (25°C/20 min). Ghafoor et al. (2020) investigated the phytochemical composition of chia seeds roasted at 90°C, 120°C, 150°C, and 180°C. Their analysis revealed that these seeds contained a variety of phenolic acids, including gallic acid, 3,4‐dihydroxybenzoic acid, 1,2‐dihydroxybenzene, caffeic acid, chlorogenic acid, cinnamic acid, and rosmarinic acid, as well as flavonoids such as quercetin, kaempferol, rutin trihydrate, genistein, myricetin, and isorhamnetin, with concentrations ranging from 4.83 to 85.49 mg/100 g depending on the compound. The study demonstrated that roasting temperature significantly affects the retention and concentration of these bioactive compounds, highlighting their contribution to the antioxidant potential and nutraceutical value of chia seeds. A previous study reported that quercetin, chlorogenic acid, and caffeic acid were present at levels of 0.17 μg/g, 4.68 μg/g, and 30.89 μg/g, respectively (Coelho and Salas‐Mellado 2014). Analyses of chia seed extracts have revealed the presence of bioactive compounds, including chlorogenic acid, caffeic acid, quercetin, and kaempferol, at varying concentrations (Reyes‐Caudillo et al. 2008). The levels of caffeic acid detected in the chia seeds examined in the present study were in agreement with those reported in earlier investigations. These values highlight the variability of caffeic acid content across different cultivars and studies. Roasting chia seeds at 120°C for 20 min increased their caffeic (31.14 → 35.46 mg/kg) and p‐coumaric (4.17 → 5.48 mg/kg) acid contents (Ghafoor et al. 2018). UAE revealed marked differences in total phenolic content and antioxidant capacity between methanolic (149.95 ± 20.39 mg GAE/100 g; 9.90 ± 0.05 mg Trolox/100 g) and hexane extracts (60.31 ± 5.99 mg GAE/100 g; 1.11 ± 0.38 mg Trolox/100 g). Significant levels of gallic, caffeic, chlorogenic, ferulic, and rosmarinic acids, as well as myricetin, quercetin, and kaempferol, have been identified in chia seeds (Dias et al. 2017). Similarly, many species of the genus *Salvia* are valued for their horticultural and medicinal uses, as they contain bioactive compounds such as flavonoids (myricetin, quercetin, kaempferol) and polyphenols (chlorogenic and caffeic acids) (Reyes‐Caudillo et al. 2008; Ixtaina, Nolasco, and Tomás 2011). It has shown that ultrasonic power is a critical factor influencing the efficiency of phenolic extraction (Hussam Ahmad‐Qasem et al. 2013). The greater total phenol content in roasted chia seeds compared to unroasted seeds could also result from the reaction of Maillard reaction products formed during roasting with Folin–Ciocalteu reagent, in addition to the phenols naturally present in the seed structure (Liu et al. 2020). The antioxidant activities of the seeds did not change significantly with both extraction systems. Only the antioxidant activities of roasted chia seeds extracted at 25°C and 40°C for 20 min were found to be slightly higher than those extracted for 40 min. The differences in phenolic compound contents between water bath and ultrasound‐assisted extracted raw and roasted chia seed samples may be attributed to variations in extraction efficiency, processing conditions, and structural changes occurring during roasting. In the present study, ultrasound‐assisted extraction resulted in differences in phenolic recovery compared with conventional water bath extraction, which is consistent with previous findings indicating that ultrasonic power enhances phenolic extraction by promoting cavitation, improving solvent penetration, and disrupting plant cell structures (Carcel et al. 2012; Hussam Ahmad‐Qasem et al. 2013; Priego‐Capote and Luque De 2004). Previous studies have reported the presence of important phenolic compounds in chia seeds, including caffeic acid, chlorogenic acid, quercetin, and kaempferol, although their concentrations varied considerably depending on cultivar, environmental conditions, and analytical procedures (Reyes‐Caudillo et al. 2008; Coelho and Salas‐Mellado 2014). For example, Coelho and Salas‐Mellado (2014) reported quercetin, chlorogenic acid, and caffeic acid concentrations of 0.17, 4.68, and 30.89 μg/g, respectively, whereas the caffeic acid levels obtained in the present study were comparable with previous reports, confirming the variability of phenolic composition among different chia samples. Furthermore, roasting treatment can modify phenolic availability by inducing thermal degradation of some compounds while simultaneously increasing extractable phenolics through cell wall disruption and the formation of Maillard reaction products. Similar results were observed by Ghafoor et al. (2018), who demonstrated that roasting chia seeds at 120°C for 20 min increased caffeic acid and p‐coumaric acid contents from 31.14 to 35.46 mg/kg and from 4.17 to 5.48 mg/kg, respectively. In addition, Liu et al. (2020) suggested that the higher total phenolic content observed in roasted seeds may be partially related to the interaction of Maillard reaction products with the Folin–Ciocalteu reagent. Therefore, the differences observed between raw and roasted chia extracts obtained by water bath and ultrasound‐assisted extraction can be explained by the combined effects of extraction technique, ultrasonic energy, thermal processing, and changes in the chemical accessibility of phenolic compounds within the chia seed matrix. Overall, the phenolic compounds in chia seeds increased compared to the control when extracted using both methods at 25°C and 40°C for 20 and 40 min. Ultrasonic and water bath extraction of raw chia seeds resulted in significant increases in the concentrations of gallic acid, 3,4‐dihydroxybenzoic acid, catechin, caffeic acid, syringic acid, rutin, *p‐*coumaric acid, and kaempferol compared to the control. Ultrasonic and water bath extraction of raw chia seeds significantly increased the accumulation of phenolic compounds such as gallic acid, 3,4‐dihydroxybenzoic acid, catechin, caffeic acid, syringic acid, rutin, p‐coumaric acid, and kaempferol compared with the control group. This increase is mainly attributed to the disruption of the seed matrix, enhanced cell wall permeability, and improved mass transfer, which facilitate the release of bound phenolic compounds. Ultrasonic cavitation promotes cellular breakdown, while water bath heating improves phenolic solubility and diffusion, resulting in higher recovery of bioactive compounds (Vilkhu et al. 2008; Chemat et al. 2017). Ultrasound‐assisted extraction (UAE) improves the recovery of phenolic compounds through acoustic cavitation and enhanced mass transfer. Acoustic cavitation involves the formation and collapse of microbubbles, generating localized high pressure, temperature, shock waves, and microjets that disrupt plant cell structures and facilitate the release of intracellular phenolics. Ultrasound‐induced microstreaming also reduces the boundary layer around particles, accelerating solvent penetration and diffusion. Because these mechanical effects enhance extraction efficiency, UAE can operate at lower temperatures, minimizing thermal degradation, oxidation, and polymerization of heat‐sensitive phenolic compounds. Consequently, UAE provides higher preservation of bioactive compounds compared with conventional high‐temperature extraction methods (Vinatoru 2001; Chemat et al. 2017). Similar effects of extraction conditions on increasing phenolic compound recovery have been reported in plant materials (Dai and Mumper 2010). Notably, both raw and roasted chia seeds demonstrated elevated levels of the predominant phenolic compounds across both extraction methods, indicating that these techniques effectively enhance the phenolic profile and potential bioactivity of chia seeds. Additionally, the amounts of phenolic compounds detected in roasted chia seeds were increased when compared to raw chia seeds after ultrasonic and water bath extraction. The highest phenolic compounds in raw chia seeds eluted with ultrasonic bath extraction were observed at 25°C/20 min and 25°C/40 min extraction temperatures, while the highest extraction times for water bath extraction were 25°C/20 min and 40°C/40 min. The phenolic compounds in raw chia seeds were higher when extracted using the ultrasonic bath system compared to the water bath extraction system. Conversely, for roasted chia seeds, the phenolic compounds were higher when extracted with the water bath system than with the ultrasonic bath system. Additionally, the levels of gallic acid, caffeic acid, rutin, *p*‐coumaric acid, and resveratrol in roasted chia seeds extracted using the water bath system at various temperatures and durations decreased compared to the control. This decrease may have been due to the agitation speed of the water bath system and the ambient temperature.

### 3.3 The Fatty Acid Profile of Raw and Roasted Chia Seed Oils

The fatty acid compositions of raw and roasted chia seed oils extracted with ultrasonic and water bath systems at different temperatures and times are given in Table 4. The predominant fatty acids in raw and roasted chia seed oils extracted with both systems were linolenic, linoleic, and oleic acid. The oleic acid content of raw chia oils obtained using ultrasonic and water bath methods ranged from 9.84% (25°C/20 min) to 10.27% (control) and from 10.12% (control) to 10.36% (25°C/20 min), respectively. Similarly, the linoleic acid content of oils obtained from raw chia seeds via the ultrasonic bath method was found to be between 18.08% (25°C/40 min) and 19.25% (25°C/20 min), while linoleic acid levels in the oils from raw seeds were measured between 18.15% (25°C/40 min) and 18.42% (40°C/20 min). Linolenic acid amounts of the oils obtained from raw chia seeds treated by ultrasonic and water bath extraction systems were detected to be between 68.91% (40°C/40 min) and 70.20% (25°C/40 min) to 69.15% (40°C/40 min) and 69.97% (control), respectively. In addition, oleic acid amounts of the oils obtained from roasted chia seeds treated by ultrasonic and water bath extraction systems were assayed to be between 9.90% (25°C/40 min) and 10.15% (25°C/20 min) to 9.92% (control) and 10.03% (40°C/40 min), respectively. The total fatty acid percentages detected under the conditions of 25°C for 40 min account for only approximately 90% of the total fatty acid composition. The remaining 10% may be attributed to undetected fatty acid components, minor fatty acid derivatives, measurement limitations, and analytical losses that can occur during sample preparation and instrumental analysis. In addition, some fatty acids may be present at very low concentrations below the detection or quantification limits of the analytical method. Therefore, the missing percentage does not necessarily indicate the absence of fatty acids but may reflect the inherent uncertainties and limitations of the analytical procedure. Also, linolenic acid amounts of the oils extracted from roasted chia seeds treated by ultrasonic and water bath systems were identified to be between 69.32% (control) and 69.71% (40°C/20 min) to 69.46% (25°C/40 min) and 69.81% (40°C/40 min), respectively. Depending on the extraction and seed types, the fatty acid amounts of other extracted oils were found to be below 5.63%. In general, the oil contents of raw and roasted chia seeds increased at 40°C/40 min for both extraction methods, while increases and decreases were observed at 25°C/20 min compared to the control. In general, the increase in oil content of both raw and roasted chia seeds after extraction at 40°C for 40 min may be attributed to the enhanced release of lipids from the seed matrix due to prolonged extraction time and increased temperature. Higher temperatures can improve solvent penetration, reduce oil viscosity, and promote the disruption of cell structures, thereby facilitating the transfer of triglycerides and other lipid components into the extracted oil phase. However, the variations (increases and decreases) observed at 20°C for 20 min compared with the control group may be related to insufficient extraction energy and the heterogeneous distribution of lipids within the seed structure. At lower temperatures and shorter extraction periods, the extraction process may not completely disrupt the cellular matrix, resulting in variable oil recovery depending on seed composition, particle size, and the availability of surface lipids. Additionally, roasting can modify the cellular structure of chia seeds by inducing thermal degradation of cell walls and changes in lipid–protein interactions, which may either enhance or limit oil release depending on the severity of the treatment. Similar effects of temperature and extraction time on oil yield and lipid recovery have been reported for oilseeds, where moderate increases in extraction temperature and duration improved lipid extraction efficiency, whereas insufficient conditions caused inconsistent recovery values (Reyes‐Caudillo et al. 2008; Ixtaina, Martínez, et al. 2011; Azadmard‐Damirchi et al. 2010). The predominant fatty acid compositions of oils obtained from raw and roasted chia seeds extracted with ultrasonic and water bath extraction systems were the same, but some differences were observed in their amounts. The oleic acid content of oils extracted from raw chia seeds using the ultrasonic bath system decreased compared to the control, whereas the oleic acid content of oil extracted with the water bath system showed a slight increase. Ultrasonic treatment of raw chia seeds may reduce oleic acid content compared with controls because cavitation generates localized heat and reactive species that can promote lipid oxidation and fatty acid degradation (Pingret et al. 2013). In contrast, water bath extraction provides gentler thermal conditions, which may enhance oil release without significant alteration of oleic acid composition (Mello et al. 2017). In contrast, the linolenic acid content of oil from raw chia seeds extracted with the ultrasonic bath system varied depending on the temperature and duration of extraction, while the linolenic acid content of oil extracted with the water bath system decreased slightly compared to the control. For oils extracted from roasted chia seeds, both ultrasonic and water bath methods showed a decrease in linoleic acid content as extraction temperature and time increased, while linolenic acid content generally increased, except in the water bath extract at 25°C for 40 min. The decrease in linoleic acid content and the increase in linolenic acid content with longer extraction time and higher temperature may be related to differences in fatty acid stability, oxidation susceptibility, and extraction efficiency. Extended exposure to heat and oxygen can accelerate the degradation of linoleic acid, while longer extraction may improve the release of linolenic acid from the chia seed matrix. The exception observed in the water‐bath extraction at 25°C for 40 min may be due to mild extraction conditions that limited oxidation and resulted in different mass transfer behavior, leading to a temporary variation in fatty acid composition. Similar effects of extraction conditions on PUFA profiles have been reported by Shahidi and Zhong (2010) and Martínez et al. (2012). Additionally, the oleic acid content of roasted chia seed oils varied according to the extraction method, temperature, and duration relative to the control. These changes are likely due to the breakdown of double bonds in unsaturated fatty acids caused by heat and oxygen during processing. The extraction conditions that produced the highest oleic and linoleic acid concentrations in water bath‐extracted roasted chia seed oil were 40°C for 40 min. Overall, neither the extraction method nor the roasting conditions significantly altered the oils' fatty acid composition, although minor variations were noted in certain fatty acids. Ghafoor et al. (2020) investigated the fatty acid profile of oils obtained from chia seeds subjected to roasting at 90°C, 120°C, 150°C, and 180°C, observing a general decrease in fatty acid content with increasing temperature. The chia seed oil contained 6.48%–7.08% palmitic acid, 2.69%–2.97% stearic acid, 9.94%–10.09% oleic acid, 19.47%–20.57% linoleic acid, and 58.64%–59.84% linolenic acid. Similarly, Özcan et al. (2019a) reported that chia oils roasted in a microwave at varying power levels exhibited palmitic, stearic, oleic, linoleic, and linolenic acid contents of 7.09%–8.35%, 1.81%–2.67%, 9.18%–10.08%, 19.21%–21.17%, and 66.84%–68.71%, respectively. In a separate study, Sargi et al. (2013) documented the fatty acid composition of chia seed oil as 5.8% palmitic acid, 2.4% stearic acid, 6.1% oleic acid, 17.4% linoleic acid, and 54.4% linolenic acid. Furthermore, Hatamian et al. (2020) reported a linoleic acid content of 58.5% in chia seed oil. Roasting at 175°C for 30 min can be considered a controlled heat treatment that improves flavor, texture, and microbial safety while limiting excessive degradation of polyunsaturated fatty acids (PUFAs). Although PUFAs are sensitive to oxidation at high temperatures, their stability depends on factors such as heating time, oxygen exposure, and the presence of natural antioxidants. A moderate roasting duration helps minimize lipid oxidation compared with longer or more intense thermal treatments, allowing acceptable retention of PUFA content (Choe and Min 2006; Zamora and Hidalgo 2001).

**TABLE 4:** Fatty acid composition of the oils of raw and roasted chia seeds extracted by ultrasonic and water bath methods.

Fatty acids (%) for raw chia seed oil	Ultrasonic bath				
	Control	25°C/20 min	40°C/20 min	25°C/40 min	40°C/40 min
Stearic	0.80 ± 0.02b	0.73 ± 0.01d	0.76 ± 0.01 cd	0.77 ± 0.05c	0.92 ± 0.02a
Oleic	10.27 ± 0.08a	9.84 ± 0.03e	10.10 ± 0.06b	9.91 ± 0.07d	10.07 ± 0.02c
Linoleic	18.43 ± 0.03c	19.25 ± 0.01a	18.19 ± 0.00d	18.08 ± 0.07e	19.04 ± 0.03b
Arachidic	0.22 ± 0.00b	0.23 ± 0.00a	0.23 ± 0.00a	0.23 ± 0.01a	0.23 ± 0.01a
Linolenic	69.46 ± 0.06c	69.16 ± 0.02d	69.92 ± 0.04b	70.20 ± 0.05a	68.91 ± 0.01e
Behenic	0.82 ± 0.02b	0.80 ± 0.01d	0.81 ± 0.02c	0.81 ± 0.01c	0.83 ± 0.02a

Fatty acids (%)	Water‐bath				
	Control	25°C/20 min	40°C/20 min	25°C/40 min	40°C/40 min
Stearic	0.80 ± 0.03b	0.80 ± 0.04b	0.77 ± 0.02d	0.78 ± 0.01c	0.81 ± 0.05a
Oleic	10.12 ± 0.07d	10.36 ± 0.09a	10.29 ± 0.00b	10.17 ± 0.08c	10.35 ± 0.06a
Linoleic	18.37 ± 0.06c	18.33 ± 0.03d	18.42 ± 0.01a	18.15 ± 0.04e	18.39 ± 0.10b
Arachidic	0.22 ± 0.00b	0.23 ± 0.02a	0.21 ± 0.01c	0.21 ± 0.01c	0.22 ± 0.01b
Linolenic	69.97 ± 0.32a	69.69 ± 0.06c	69.58 ± 0.11d	69.86 ± 0.11b	69.15 ± 0.23e
Behenic	0.82 ± 0.02b	0.60 ± 0.02d	0.73 ± 0.15c	0.83 ± 0.01a	0.83 ± 0.02a

Fatty acids (%) for roasted chia seed oil	Ultrasonic bath				
	Control	25°C/20 min	40°C/20 min	25°C/40 min	40°C/40 min
Stearic	0.72 ± 0.02c	0.69 ± 0.01d	0.67 ± 0.04e	0.76 ± 0.06b	0.82 ± 0.08a
Oleic	10.01 ± 0.08c	10.15 ± 0.03a	10.10 ± 0.13b	9.90 ± 0.09d	9.91 ± 0.06d
Linoleic	18.70 ± 0.05a	18.49 ± 0.03b	18.27 ± 0.03d	18.45 ± 0.03c	18.50 ± 0.03b
Arachidic	0.24 ± 0.00b	0.25 ± 0.01a	0.24 ± 0.01b	0.23 ± 0.01c	0.23 ± 0.01c
Linolenic	69.32 ± 0.04d	69.39 ± 0.00c	69.71 ± 0.06a	69.67 ± 0.03b	69.61 ± 0.06b
Behenic	5.63 ± 6.55a	1.03 ± 0.00b	1.00 ± 0.00c	0.99 ± 0.02d	0.93 ± 0.00e

Fatty acids (%)	Water‐bath				
	Control	25°C/20 min	40°C/20 min	25°C/40 min	40°C/40 min
Stearic	0.75 ± 0.07c	0.72 ± 0.04d	0.78 ± 0.01a	0.77 ± 0.01b	0.77 ± 0.06b
Oleic	9.92 ± 0.06e	10.02 ± 0.04b	9.99 ± 0.02c	9.94 ± 0.01d	10.03 ± 0.06a
Linoleic	18.42 ± 0.06b	18.33 ± 0.00c	18.30 ± 0.02d	18.62 ± 0.03a	18.28 ± 0.00e
Arachidic	0.24 ± 0.01a	0.22 ± 0.01c	0.23 ± 0.00b	0.23 ± 0.00b	0.23 ± 0.01b
Linolenic	69.66 ± 0.11d	69.72 ± 0.03b	69.70 ± 0.03c	69.46 ± 0.05e	69.81 ± 0.03a
Behenic	1.02 ± 0.02a	0.99 ± 0.01c	1.00 ± 0.01b	0.98 ± 0.00d	0.88 ± 0.02e

*Note:* Values within each row followed by different letters are significantly different at *p* < 0.05.

### 3.4 Oxidizability, Oleic, and Linoleic Desaturation Ratiosof the Oils Extracted From Chia Seeds Sonicated at Different Temperatures and Times

Calculation Lipid index values of raw and roasted chia seeds obtained by ultrasonic and water bath extraction methods are given in Table 5. The oxidizability values of raw and roasted chia seeds extracted by ultrasonic and water bath extraction at different temperatures and times were determined to be between 16.95 (40°C–40 min) and 17.12 (25°C–40 min) to 16.93 (40°C–40 min) and 17.10 (control), respectively. The oxidizability values of roasted chia seed oils obtained using the water extraction system ranged from 17.02 (25°C–40 min) to 17.06 (40°C–40 min), compared to 17.04 for both 40°C–20 min and 40°C–40 min, and the control and 25°C–20 min samples. Oleic desaturation ratios of raw and roasted chia seed oils obtained via the ultrasonic bath system were measured between 0.895 (control) and 0.900 (25°C–20 min) for raw oils, and 0.896 (25°C–20 min) and 0.899 (25°C–40 min and 40°C–40 min) for roasted oils. Similarly, oleic desaturation ratios for oils obtained with the water bath system ranged from 0.894 (40°C–40 min) to 0.897 (control) for raw oils, and from 0.894 (40°C–20 min) to 0.899 (control and 40°C–20 min) for roasted oils. Linoleic desaturation values of raw chia seed oils obtained by ultrasonic and water bath systems were between 0.782 (25°C–20 min) and 0.795 (25°C–40 min) and 0.790 (40°C–40 min) and 0.794 (25°C–40 min), respectively. For roasted chia seed oils, linoleic desaturation values were measured between 0.788 (control) and 0.792 (40°C–20 min) for the ultrasonic system, and 0.788 (25°C–40 min) and 0.792 (25°C–20 min, 40°C–29 min, and 40°C–40 min) for the water bath system. The oxidizability stability of the oil obtained from raw chia seeds extracted with ultrasonic and water bath systems at 40°C for 40 min was slightly higher than that of other oils. The slightly higher oxidative stability of chia seed oil extracted by ultrasonic‐assisted and water‐bath methods at 40°C for 40 min may be related to the preservation of natural antioxidant compounds during the mild extraction process. Although chia oil contains high levels of oxidation‐sensitive polyunsaturated fatty acids, especially α‐linolenic acid, its tocopherols and phenolic compounds can inhibit free radical formation and delay lipid oxidation (Ixtaina, Nolasco, and Tomás 2011; Shahidi and Zhong 2010). The moderate extraction temperature reduces thermal degradation of these bioactive compounds, while ultrasound improves cell disruption and oil recovery without excessive heat exposure, contributing to better oxidative stability (Chemat et al. 2017). Therefore, the combination of efficient extraction and preservation of endogenous antioxidants may explain the slightly higher oxidation resistance of chia seed oil compared with some other vegetable oils (Capitani et al. 2012). Roasting partially reduced the oxidizability value, ODR, and LDR values of the oils compared to those of raw chia oils. The generally low oleic and linoleic desaturation rates suggest that chia oil may have health benefits. Mondal et al. (2010) reported average values of 0.5 for ODR and 0.01 for LDR. In a more recent study, El‐Beltagi et al. (2022) found that the oil extracted from sesame seeds roasted using various methods exhibited oxidizability values ranging from 4.97 to 5.06, ODR values between 0.5143 and 0.5172, and LDR values from 0.0115 to 0.0136.

**TABLE 5:** Oxidizability, oleic and linoleic desaturation ratios of the oils of raw and roasted chia seeds extracted by ultrasonic and water bath methods.

Indices for raw chia seed oils	Ultrasonic bath				
	Control	25°C/20 min	40°C/20 min	25°C/40 min	40°C/40 min
Oxidizability value (Cox)	17.00	17.01	17.07	17.12	16.95
Oleic desaturation ratio (ODR)	0.895	0.900	0.897	0.899	0.897
Linoleic desaturation (LDR)	0.790	0.782	0.794	0.795	0.784

Indices	Water bath				
	Control	25°C/20 min	40°C/20 min	25°C/40 min	40°C/40 min
Oxidizability value (Cox)	17.10	17.03	17.02	17.06	16.93
Oleic desaturation ratio (ODR)	0.897	0.895	0.895	0.896	0.894
Linoleic desaturation (LDR)	0.792	0.792	0.791	0.794	0.790

Indices for roasted chia seed oils	Ultrasonic bath				
	Control	25°C/20 min	40°C/20 min	25°C/40 min	40°C/40 min
Oxidizability value (Cox)	16.99	16.99	17.04	17.00	17.04
Oleic desaturation ratio (ODR)	0.898	0.896	0.897	0.899	0.899
Linoleic desaturation (LDR)	0.788	0.790	0.792	0.791	0.790

Indices	Water bath				
	Control	25°C/20 min	40°C/20 min	25°C/40 min	40°C/40 min
Oxidizability value (Cox)	17.04	17.05	17.04	17.02	17.06
Oleic desaturation ratio (ODR)	0.899	0.898	0.894	0.899	0.898
Linoleic desaturation (LDR)	0.791	0.792	0.792	0.788	0.792

## 4 Study Limitations

This study has some limitations that should be considered when interpreting the findings. The effects of roasting and extraction conditions were evaluated under specific experimental settings, and variations in processing parameters may lead to different outcomes. Additionally, the study focused on selected bioactive compounds, fatty acid profile, and lipid quality; therefore, other potentially relevant nutritional and functional properties were not assessed. Further studies involving different processing conditions and broader analytical approaches are recommended to provide a more comprehensive understanding of chia seed extract characteristics.

## 5 Conclusions

The applied extraction type, temperature, and times had an effect on the oil content and bioactive properties of raw and roasted chia seeds. The results demonstrate that processing intensity critically governs the interplay between oil content and antioxidant constituents in chia seeds. The highest total phenol content was detected in roasted chia seeds extracted with ultrasonic and water bath systems. The phenolic compounds of roasted chia seeds were generally higher than those of raw chia seeds. In general, the predominant phenolic compounds identified in roasted chia seeds were similar to those in raw chia seeds, although there were some differences. The total phenol and flavonoid contents of roasted chia seeds were higher than those of unroasted chia seeds. Additionally, total phenol and flavonoid contents of chia seeds increased compared to the control group using both extraction systems (ultrasonic and water bath). As can be seen, the increase in phenolic compounds of raw and roasted chia seeds with both extractions was observed in the predominant phenolic compounds.

## Author Contributions

**Mehmet Musa Özcan:** investigation, formal analysis, writing – original draft, writing – review and editing. **Elfadıl E. Babiker:** methodology, data curation, writing – review and editing. **Mahmoud Younis:** investigation, writing – review and editing. **Fahad AlJuhaimi:** validation, data curation, writing – review and editing. **Kashif Ghafoor:** writing – review and editing, data curation, conceptualization. **Nurhan Uslu:** formal analysis. **Isam A. Mohamed Ahmed:** conceptualization, methodology, visualization, writing – review and editing. **Belal M. Mohammed:** validation, data curation, writing – review and editing.

## Funding

This study was funded (ORF‐2026‐1573) by King Saud University, Riyadh, Saudi Arabia.

## Disclosure

*Author Approval and Responsibility Statement*: All authors have read and approved the final version of the manuscript. [Corresponding Author/Manuscript Guarantor] had full access to all of the data in this study and takes complete responsibility for the integrity of the data and the accuracy of the data analysis.

## Ethics Statement

This study on *extraction* was conducted exclusively using plant material (Chia seed) and did not involve any human participants, human data, animals, or animal‐derived experimental procedures. Therefore, ethical approval from a human or animal ethics committee was not required for this research. All experimental procedures were carried out in accordance with relevant institutional and scientific guidelines for plant‐based studies.

## Conflicts of Interest

The authors declare no conflicts of interest.

## Data Availability

The results of this study are available with permission from the authors.
